# Effects of Perceived Parent–Child Relationships and Self-Concept on Creative Personality among Middle School Students

**DOI:** 10.3390/bs14010058

**Published:** 2024-01-16

**Authors:** Hyesung Park, Sungyeun Kim

**Affiliations:** Graduate School of Education, Incheon National University, 309Ho 15Hokwan Academiro 119, Incheon 22012, Republic of Korea; bark0115@snu.ac.kr

**Keywords:** adolescence, creativity education, creative personality, parent–child relationships, self-concept

## Abstract

This study investigated the impact of perceived parent–child relationships on creative personality in middle school students according to self-concept, focusing on environmental (parent–child relationships) and individual (self-concept) factors that can influence these students’ creative personalities. To this end, this study verified the moderation effect using sixth-year data (third year of middle school students) from the Seoul Longitudinal Educational Study 2010 Panel, utilizing SPSS 26.0 and the PROCESS macro. The results revealed that the self-concept of middle school students moderated the influence of parent–child relationships on creative personality. Specifically, it was found that as the level of self-concept increased above the average the positive relationship between parent–child relationships and creative personality strengthened. Based on the study’s findings, theoretical and practical suggestions for creating a home and educational environment to promote creativity during adolescence were discussed.

## 1. Introduction

As creativity is a multifaceted and integrated attribute, it is important to consider both the social-environmental factors surrounding individuals and individual factors together when understanding creativity. Amabile [1] proposed the Componential Theory of Creativity, which emphasized the importance of these social environmental elements in creativity, helping to understand how situational contexts can influence creativity development. Therefore, this study aimed to verify the influence of environmental factors (parent–child relationships) and individual factors (self-concept) on the creative personalitiy of middle school students. Recognizing that creativity is not a stable trait but is based on the interaction between social, environmental, and individual factors assumes that creativity can be developed [2], which is significant in providing specific interventions for developing creativity.

Although scholars have defined creative personality in various ways, curiosity, openness, imagination, playfulness, risk-taking propensity, and originality are commonly utilized as being representative of creative personality [3]. Such creative personality traits can help in breaking away from conventional thinking, broadening the scope of thought, activating the association of disparate ideas, and positively influencing creativity manifestation [4,5]. Generally, creative personality shows a declining pattern from the upper grades of elementary to middle school, with the decline being particularly prominent in middle school compared to elementary school [6]. Therefore, it is imperative to explore the factors influencing creative personalities, which can decrease during middle school.

The creative personality traits, particularly those observed during adolescence, hold significance due to their ability to affect subsequent creative performance through divergent thinking [7,8]. Therefore, to provide concrete implications for fostering creative personality and creativity during adolescence, it is essential to identify environmental factors that can influence these personality factors and individual factors that can moderate these relationships. Accordingly, this study focused on the perceived parent–child relationship as an environmental factor that influences the creative personality of middle school students and self-concept as an individual factor that can moderate this relationship.

One of the major environmental factors influencing the development of creative personalities is parents. As significant others who interact closely with adolescents over a long period, parents are key environmental factors that should be considered in understanding the development of creativity during adolescence [6,8]. In this parent–child relationship, the impact varies depending on how the child perceives the actual parenting style [9]. Consequently, understanding the child’s thoughts and interpretations of their relationship with their parents is more important than the parents’ perceptions of their attitudes.

Parents maintaining a warm, encouraging, and positive relationship can foster the development of creative personality traits such as curiosity, openness, sensitivity, imagination, and independence in their children [10]. Specifically, parents nurturing their children’s curiosity, divergent thinking, and imagination within a close relationship have been found to positively influence creativity development [11]. Kim et al. [6], who explored longitudinal changes and predictors of creativity development in adolescence, analyzed creativity changes from the fourth grade in elementary school to the third grade in middle school, considering the influence of parents, teachers, and peer relationships as environmental factors. They found that perceived creativity in students gradually decreased over time, especially during middle school, where the decrease was substantial. During this process, perceiving a positive relationship with parents, teachers, and peers mitigated this declining trend in creativity. Such findings suggested that satisfying a sense of belonging and relational needs within a positive parent–child relationship has a positive impact on creativity manifestation [12,13].

Meanwhile, while adolescent students are still under their parents’ influence, having just recently shifted their primary living space from home to institutional environments such as school [14], parents’ direct influence tends to decrease as the child ages and spends more time in various social spaces [15]. Therefore, when examining parent–child relationships among students in middle school or older, it is important to consider cognitive and motivational characteristics that have developed within the individual’s developmental process rather than just addressing the isolated impact of parents. However, previous studies that have explored the relationship between parental factors and children’s creativity have mainly focused on examining the isolated effects of parenting attitudes or the parent–child relationship on the development of creativity [6,16,17]. Some studies have also emphasized mechanisms that mediate the relationship between parental factors and creativity [12,18]. Thus, there is still a lack of research that explores moderating variables to enhance the relationship between parents and children’s creativity.

Self-concept generally refers to one’s cognitive assessment of their characteristics or abilities. It can be categorized into sub-factors such as general self-concept, which represents overall beliefs about oneself; academic self-concept, which is related to beliefs about academic abilities; and social self-concept, which is related to beliefs about social abilities [19]. However, this study focuses on general self-concept, which involves overall evaluation and beliefs about oneself. Self-concept is similar to self-efficacy and self-esteem, but self-efficacy and self-esteem differ conceptually from self-concept, primarily in that they include emotional responses and evaluations of oneself [20]. However, they are generally used interchangeably to some extent due to their common basis in self-perception, which is rooted in competence and evaluation of oneself.

Rather than being a stable attribute, self-concept is formed through experiences and interpretations of one’s environment [21]. In other words, self-concept is not a fixed trait but develops based on relationships and interactions with others. Even during adolescence, individuals continue to be influenced by their parents—a primary environment. Positive parenting attitudes and relationships perceived by the child have been shown to correlate with self-recognition of their value and existence [22,23,24]. Conversely, children with a high self-concept tend to perceive positive interactions and relationships with their parents [23]. Through this, it can be expected that students with high self-concepts in the context of positive parent–child relationships are likely to achieve positive outcomes in academic settings.

Forming a positive self-concept during adolescence is crucial for the development of creativity and a creative personality. According to several theories related to creativity [1,25], motivational resources such as self-concept and self-efficacy can act as internal forces for creative ideas and activities. Amabile and Pillemer [26] stated that their Componential Theory of Creativity, a socio-psychological approach to creativity, proposed that the interaction between social environmental factors and an individual’s intrinsic motivation can lead to the manifestation of creativity. Empirical studies have also confirmed the influence of self-concept on creativity manifestation. For example, Felker and Treffinger [27] examined the relationship between self-concept, divergent thinking, and attitudes toward problem-solving in elementary school students. Students with a high self-concept demonstrated higher scores in fluency, originality, and flexibility than those with lower self-concepts. Elementary students with high self-efficacy also exhibited higher levels of creativity [28,29]. This suggested a close connection between self-concept and creativity, where individuals with a positive self-view tend to tackle challenges with confidence rather than anxiety, leading to the development of creative problem-solving abilities [30]. Similarly, elementary students with high self-efficacy demonstrated higher levels of creativity [28]. Based on these findings, it can be speculated that students with a high self-concept are likely to perceive greater emotional support and positive interactions with their parents, which, in turn, could positively influence the development of their creative personalities.

In summary, parents are significant environmental factors influencing the development of creative personality in adolescent students, and a positive perception of the parent–child relationship is associated with the development of creative personality. Specifically, it is reported that a higher self-concept, representing beliefs and cognitive evaluations about oneself that can be influenced by the surrounding environment, may amplify the positive impact of perceived parent–child relationships on creative personality. However, most existing studies have limitations in not examining moderating variables that could flexibly affect the relationship between parents and their child’s development. Therefore, this study aimed to investigate the influence of self-concept, which may moderate the relationship between parent–child relationships and creative personality. Through this, it would be possible to complement previous studies on adolescent creative personalities and provide practical intervention strategies for fostering creativity. The research questions for this study are as follows:

**Research Question** **1.** *What is the relationship between the perceived parent–child relationship, self-concept, and creative personality in middle school students?*

**Research Question** **2.** *How does the impact of perceived parent–child relationships on creative personality vary depending on self-concept in middle school students?*

## 2. Research Methodology

### 2.1. Participants

All variables in this study were analyzed using items included in the Seoul Education Longitudinal Study (SELS) 2010. The Seoul Education Longitudinal Study (SELS) 2010 aimed to systematically understand how students in Seoul, Korea, through their experiences of Seoul Metropolitan Office of Education policies and school education, grow and evolve. Sixth-year data (third year of middle school) from the Seoul Longitudinal Education Study (SELS) 2010 Elementary School Panel were used, which comprised 3673 middle school student participants. However, those who did not respond to all variables necessary for the analysis of this study were excluded, resulting in a final sample of 3631 for analysis. Specifically, the number of male students utilized in the final analysis was 1840 (50.7%), while the number of female students was 1791 (49.3%).

### 2.2. Assessments

#### 2.2.1. Creative Personality

Nine items measuring creativity from the SELS 2010 were used as the tool to measure creative personality. To measure creativity personality, SELS 2010 utilized the self-reported Integrated Creativity Scale developed by Byungki Park and Hyunsuk Kang [31]. It assessed creative personality, with three items each for the following factors: curiosity under creative motives (e.g., “I can’t just pass by when I see something new”); originality under creative attitudes (e.g., “Whenever I do something, I want to create something of my own”); knowledge-seeking under creative abilities (e.g., “I diligently search for information about what I need”). These factors are measured on a 5-point Likert scale (1: not at all, 5: very much so). This study used the mean of the nine items included in the creativity factor as the measure of creative personality. The reliability (Cronbach’s) of the scale was 0.92.

#### 2.2.2. Parent–Child Relationship

Nine items measuring the student’s perceived parent–child relationship from the SELS 2010 were utilized (e.g., “They make an effort to spend considerable time with me”), which were measured on a 5-point Likert scale (1: not at all, 5: very much so). This study interpreted the student’s perceived parent–child relationship to signify parental emotional support based on the composition of the items and the content of the measures from the SELS 2010. The reliability (Cronbach’s) of the scale was 0.94.

#### 2.2.3. Self-Concept

Five items measuring students’ self-concept from the SELS 2010 were used. The items measuring self-concept included concepts such as “I think I am a person of worth” and “I have a positive attitude toward myself” and were measured on a 5-point Likert scale (1: not at all, 5: very much so). The reliability (Cronbach’s) of the scale was 0.94.

#### 2.2.4. Residential Area

In addition to the main variables of interest in this study, the residential area was utilized as a control variable, including the local environment, which is known to cause educational disparities and influence creative personalities differently [32]. The item measuring residential area was “Where do you live?” and allowed for responses from any of Seoul’s 26 regions.

### 2.3. Analysis Method

The analysis of this study was conducted using SPSS 26.0 and the PROCESS macro. Descriptive statistics and correlation analysis between variables were analyzed to understand the demographic characteristics of the research participants and check the normality of the data in this study. Subsequently, to confirm the effect of the perceived parent–child relationship on creative personality according to self-concept in middle school students, a moderation effect test was conducted using Hayes’s PROCESS macro (model 1). When the interaction term was significant, a simple slope analysis was conducted to confirm the relationship between the parent–child relationship and creative personality at each level of self-concept. The significance of the statistical values was verified at the *p* < 0.05 level.

## 3. Results

### 3.1. Descriptive Statistics and Correlation Analysis

The descriptive statistics and correlation analysis results for variables at each measurement point are shown in Table 1. Creative personality showed a significant positive correlation with both parent–child relationships and self-concept, with the correlation with self-concept being more pronounced. In all variables, skewness was less than an absolute value of 2, and kurtosis was also less than an absolute value of 7, meeting the assumption of normality [33].

### 3.2. Effect of Perceived Parent–Child Relationship on Creative Personality According to Self-Concept in Middle School Students

To examine the effect of the perceived parent–child relationship on creative personality in middle school students according to self-concept, a regression analysis was conducted, and the results are shown in Table 2. The regression model explained creative personality significantly, with a model fit of $R^{2}$ = 0.57, *F* (4, 3626) = 445.24, *p* < 0.001. The significance of the regression coefficients indicates that the interaction between the perceived parent–child relationship and self-concept was significant in explaining creative personality (B = 0.01, *t* = 5.86, *p* < 0.001).

**Figure 1:**
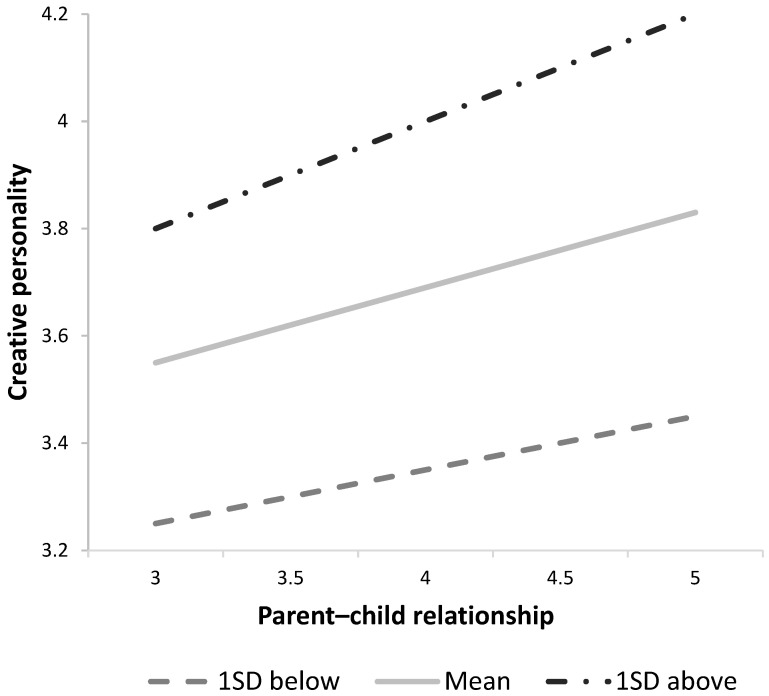
Moderation effect of self-concept.

To understand the specific nature of the interaction, a simple slope analysis was performed (see Table 3). The results of the simple slope analysis revealed that in the confidence intervals across three levels of self-concept, none included 0. This indicated a relationship where the influence of parent–child relationships on creative personality was stronger in groups perceiving higher levels of self-concept (see Figure 1). Specifically, when the middle school students’ self-concept was one standard deviation lower than the mean (B = 10, *t* = 6.14, *p* < 0.001), at the mean level of self-concept (B = 0.16, *t* = 11.82, *p* < 0.001), and one standard deviation higher than the mean, it was evident that parent–child relationships significantly influenced creative personality (B = 0.22, *t* = 12.46, *p* < 0.001).

The results showed a moderating effect at all three levels of self-concept, as all confidence intervals did not include 0. Specifically, as self-concept increased, the parent–child relationship had a positive effect on creative personalities (see Figure 1). Additionally, to determine which areas across the entire range of the moderator have an influence, the Johnson–Neyman technique was implemented. The significant regions from the analysis are shown in Table 4. It was found that the parent–child relationship had a significant effect on creative personality when the level of self-concept exceeded 2.4000, as the 95% confidence interval did not include 0.

## 4. Discussion and Conclusions

This study aimed to investigate the relationship between parent–child relationships, a key environmental factor that can influence middle school students’ creativity, with a focus on creative personality. Additionally, this study determined whether self-concept, a personal factor, moderated the relationship between perceived parent–child relationships and creative personality. To achieve this, sixth-year data from the SELS 2010 Panel were analyzed to examine the effect of the perceived parent–child relationship on creative personality as moderated by self-concept. The main research findings and discussions are as follows.

First, the perceived parent–child relationship of middle school students showed a positive impact on creative personality. This result aligned with previous studies indicating that recognizing a positive relationship with parents or positive parenting attitudes enhanced creative personality and creativity [6,12,34]. This outcome can be understood as the sense of belonging and relational needs being met through positive recognition of the relationship with parents, which positively influenced the manifestation of creative personality and creativity, such as curiosity, openness, and sensitivity [12,13]. Park et al. [12] found that when children perceived controlling parenting rather than warmth from their parents, it led to lower levels of creative personality through negative play behaviors. This suggests that in order to foster the healthy development of creative personalities, it is important to create a family atmosphere where children perceive emotional support and positive relationships with their parents, rather than a sense of control. In particular, as children grow older, parents remain a significant environmental factor that can consistently influence their creativity [10,35]. Therefore, it is important to create a positive atmosphere that can expand a child’s thinking and perspective, aiding in creative personality traits such as openness, curiosity, and imagination. For instance, engaging in frequent conversations with children about everyday matters, career aspirations, and social relationships can help them feel emotional support and affection from their parents, ensuring they receive ample love and care.

Second, the self-concept of middle school students was found to moderate the relationship between perceived parent–child relationship and creative personality. In other words, the interaction between the perceived parent–child relationship and self-concept significantly influenced creative personality, indicating that the higher a middle school student’s self-concept, the greater the impact of the perceived parent–child relationship on creative personality. This supported existing theories of creativity, suggesting that intrinsic motivation can serve as an internal force for generating creative ideas or engaging in creative activities [1,25]. According to Amabile’s Componential Theory of Creativity [1], creativity can manifest through the interaction of social environmental factors and individual internal motivation. Consistent with this, this study also showed that the relationship with parents (an environmental factor) and self-concept (an individual factor) influenced the enhancement of creativity. These results can be understood in the context of studies showing that students with a high self-concept were more likely to perceive their relationship with their parents positively [23] and exhibited a higher level of creative personality and creativity [27,28,29]. Students with a high self-concept evaluate and perceive themselves positively, possessing an active motivation to solve problems. Thus, even when encountering difficulties in everyday life, they exhibit resilience based on the perceived stable emotions and support received from their parents, demonstrating a connection to creative personality and creativity in seeking to solve problems in new ways.

Previous studies mainly explored the isolated relationship between parental factors and creativity, whereas this study examined the moderating effect of self-concept, which can develop based on relationships with others rather than being a fixed trait. In particular, as the level of self-concept rises above the average, the impact of perceived parent relationships on creative personality increases. Self-concept typically increases before adolescence, decreases during adolescence, and stabilizes with increasing age [36]. This suggests that to foster the development of creative personality during middle school, a period when self-concept is shown to decrease compared to elementary school, it can be helpful for both families and schools to provide guidance on maintaining a positive perception of self-concept. For example, parents can engage in sufficient conversations and guidance in everyday life, at home, and within the school environment to help children perceive themselves positively in terms of their values and roles. 

However, in this study, it was found that the relative impact of perceived parent–child relationships was greater than the interaction between perceived parent–child relationships and self-concept. This aligns with previous findings suggesting that even as students transition to middle school and their primary living environment shifts to school, they continued to be influenced by their parents [14]. Therefore, to foster the development of creative personality in middle school students, it is important to take a comprehensive approach that focuses not only on isolated aspects but also on enhancing both environmental factors such as parent–child relationships and personal factors such as self-concept.

Finally, this study explored the influence of parents on children’s development, taking into account variables that can lead to educational disparities based on children’s socio-economic backgrounds. This is because creativity can be influenced differentially by parents’ socio-economic status and background [37]. Therefore, it is important to note that the results regarding the impact of perceived parent–child relationships on creative personality based on self-concept in this study were examined under the assumption that the effects were consistent across students’ residential areas. This approach suggests that fostering positive relationships with parents and developing positive self-evaluations and beliefs can be beneficial for the development of creative personalities and creativity in middle school students, regardless of educational or regional disparities. 

This study is significant as it identifies the interaction between perceived parental relationships as an environmental factor and self-concept as an individual factor in the development of creative personalities in middle school students and provides relevant information for creating an environment conducive to promoting creativity in adolescents. Previous research on adolescent creativity has primarily focused on the influence of parents [6,16,17]. However, this study revealed that self-concept, a characteristic not fixed and susceptible to environmental influences, can moderate the relationship between parents and creative personality development. This allows for the provision of practical guidelines for enhancing creativity in both home and educational settings. For example, creating a home environment that provides emotional support while encouraging positive self-assessment during adolescence could be beneficial in fostering creative thinking in middle school children. Furthermore, by controlling for the influence of socio-economic background, specifically regional disparities, this study rigorously examined the main variables of interest. Conclusively, the significance of this study lies in confirming that fostering a positive self-concept during adolescence based on a positive relationship with parents during middle school, irrespective of the family’s economic status, can contribute to enhancing creativity.

The limitations of this study and recommendations for future research are as follows. Firstly, this study focused on the influence of parents on creative personality development without distinguishing between the impacts of fathers and mothers. However, due to the nature of parent–child relationships, children may perceive individual influences from their fathers and mothers [38,39], which could potentially have differential effects on creative personality development. Future research needs to investigate whether the relationships among the variables examined in this study are influenced differently by the genders of the parents. Secondly, due to the nature of panel data, there were certain limitations in measuring variables according to the researcher’s intent. For instance, in the case of creativity, beyond the explored creative personality in this study, objective indicators such as creative performance could have been measured. Therefore, it is necessary in future studies to examine whether the relationships identified in this study also apply to objective indicators of creativity. Also, the sixth-year SELS 2010 panel data used in this study did not include information about students’ genders. As previous research reported gender differences in the relationship between parents and creative personality and creativity [40,41], empirical studies should investigate how the relationships among variables may vary by students’ gender in the future. Lastly, due to the limitations of measuring at specific time points in panel data, this study mainly examined the interaction effects of how middle school students perceived their relationships with parents on their creative personalities, moderated by their self-concept, at a single time point. However, in future research, it would be beneficial to explore how the relationships between the variables evolve longitudinally across different time points in order to provide a broader range of educational implications for enhancing creativity.

## Figures and Tables

**Table 1:** Descriptive statistics and correlation analysis results.

		1	2	3	4
**1**	Parent–Child Relationship	-			
**2**	Self-Concept	0.47 **	-		
**3**	Creative Personality	0.40 **	0.55 **	-	
**4**	Residential Area	0.02	−0.02	0.03 *	-
	Average	3.84	3.83	3.69	12.23
	Standard Deviation	0.77	0.80	0.70	7.10
	Minimum	1.00	1.00	1.00	1.00
	Maximum	5.00	5.00	5.00	26.00
	Skewness	−0.46	−0.27	−0.11	0.08
	Kurtosis	0.24	−0.26	0.29	−1.13

* *p* < 0.05, ** *p* < 0.01.

**Table 2:** Effect of perceived parent–child relationship on creative personality according to self-concept in middle school students.

	B	S.E.	*t*	95% CI
Parent–child relationship (X)	0.13 *	0.05	2.58	[0.03, 0.23]
Self-concept (M)	0.11 *	0.05	2.01	[0.00, 0.21]
X × M	0.01 ***	0.01	5.86	[0.05, 0.10]
Residential area	0.00 **	0.00	3.05	[0.00 0.01]
$R^{2}$	0.57			
F	445.24 ***			

* *p* < 0.05, ** *p* < 0.01, *** *p* < 0.001.

**Table 3:** Verification of simple slope according to the conditional value of self-concept.

Self-Concept	B	S.E.	*t*	95% CI
M − 1SD	0.10 ***	0.02	6.14	[0.07, 0.13]
M	0.16 ***	0.01	11.82	[0.14, 0.19]
M + 1SD	0.22 ***	0.02	12.46	[0.19, 0.26]

*** *p* < 0.001.

**Table 4:** Significant regions of moderation effect.

Self-Concept	B	S.E.	*t*	95% CI
1.0000	−0.07	0.04	−1.64	[−0.14, 0.01]
1.2000	−0.05	0.04	−1.31	[−0.12, 0.02]
1.4000	−0.03	0.03	−0.93	[−0.10, 0.04]
1.6000	−0.02	0.03	−0.49	[−0.08, 0.05]
1.8000	0.00	0.03	0.02	[−0.06, 0.06]
2.0000	0.02	0.03	0.62	[−0.04, 0.07]
2.2000	0.03	0.03	1.32	[−0.02, 0.08]
2.3559	0.05	0.02	1.96	[0.00, 0.09]
2.4000	0.05 **	0.02	2.16	[0.00, 0.10]
2.6000	0.07 ***	0.02	3.15	[0.03, 0.11]
2.8000	0.08 ***	0.02	4.32	[0.05, 0.12]
3.0000	0.10 ***	0.02	5.68	[0.07, 0.13]
3.2000	0.12 ***	0.02	7.22	[0.08, 0.15]
3.4000	0.13 ***	0.01	8.85	[0.10, 0.16]
3.6000	0.15 ***	0.01	10.42	[0.12, 0.18]
3.8000	0.16 ***	0.01	11.73	[0.14, 0.19]
4.0000	0.18 ***	0.01	12.61	[0.15, 0.21]
4.2000	0.20 ***	0.02	13.04	[0.17, 0.23]
4.4000	0.21 ***	0.02	13.09	[0.18, 0.25]
4.6000	0.23 ***	0.02	12.89	[0.20, 0.27]
4.8000	0.25 ***	0.02	12.57	[0.21, 0.29]
5.0000	0.26 ***	0.02	12.19	[0.22, 0.31]

** *p* < 0.01, *** *p* < 0.001.

## Data Availability

Data can be downloaded at http://serii.re.kr (accessed on 4 March 2023).
